# Spectral Gini Index for Quantifying the Depth of Consciousness

**DOI:** 10.1155/2016/2304356

**Published:** 2016-10-20

**Authors:** Kyung-Jin You, Gyu-Jeong Noh, Hyun-Chool Shin

**Affiliations:** ^1^Department of Electronic Engineering, Soongsil University, Seoul 06978, Republic of Korea; ^2^Department of Anesthesiology and Pain Medicine, Asan Medical Center, University of Ulsan College of Medicine, Seoul 05535, Republic of Korea; ^3^Department of Clinical Pharmacology and Therapeutics, Asan Medical Center, University of Ulsan College of Medicine, Seoul 05535, Republic of Korea

## Abstract

We propose indices that describe the depth of consciousness (DOC) based on electroencephalograms (EEGs) acquired during anesthesia. The spectral Gini index (SpG) is a novel index utilizing the inequality in the powers of the EEG spectral components; a similar index is the binarized spectral Gini index (BSpG), which has low computational complexity. A set of EEG data from 15 subjects was obtained during the induction and recovery periods of general anesthesia with propofol. The efficacy of the indices as indicators of the DOC was demonstrated by examining Spearman's correlation coefficients between the indices and the effect-site concentration of propofol. A higher correlation was observed for SpG and BSpG (0.633 and 0.770, resp., *p* < 0.001) compared to the conventional indices. These results show that the proposed indices can achieve a reliable quantification of the DOC with simplified calculations.

## 1. Introduction

The depth of anesthesia (DOA) must be precisely and appropriately controlled according to the surgical procedure and the patient's medical condition. For example, inadequate anesthesia may provoke stress responses of the body such as hypertension, tachycardia, sweating, lacrimation, increased skeletal muscle tone, and spontaneous movement [1]. Tachycardia and hypertension can lead to various side effects such as a cardiovascular event. In contrast, an anesthetic agent overdose can cause hypotension, which can lead to hypoperfusion of the heart and brain in susceptible patients. Owing to the interpatient variability of the dose-response effect of anesthetic agents, the administration of an adequate amount of anesthetics and the maintenance of an appropriate DOA are challenging. Therefore, an objective and reliable method of evaluating the DOA is needed to maintain a stable level of anesthesia.

General anesthesia (GA) includes two independent components: hypnosis and analgesia [2]. Several methods of measuring the DOA are based on the changes in the autonomic nervous system, such as the degree of muscle relaxation, hemodynamics, sweating, and lacrimation [3, 4]. Methods using the heart rate variability reflect the changes in brainstem function [5, 6]. However, these parameters are poorly correlated with the cerebral cortex functions, are closely related to consciousness, and constitute poor indicators of the depth of consciousness (DOC) [7, 8]. Intraoperative awareness can occur without monitoring the DOC. Intraoperative awareness is the unexpected explicit recall of sensory perceptions during GA [9] and may occur in 0.1–0.2% of patients receiving GA [10]. Such awareness can lead to mental sequelae and posttraumatic syndrome [11]. Therefore, the parameters that monitor the DOC must focus on the electroencephalogram (EEG), which reflects the action of the cerebral cortex, of the thalamus, and of the brainstem. Many studies have attempted to develop indices for a quantitative, immediate, and continuous indicator of the DOC based on (sub)cortical electrical activities.

Information theoretical approaches, such as the spectral entropy [12–14], permutation entropy (PE) [15], and approximate entropy (AE) [16] methods, consider that the irregularity of the EEG change during anesthesia is expressed by information quantity. Although the spectral entropy approach has been clinically applied [17], these methods have the drawback that the estimation of the probability distribution, which is the theoretical basis of these methods, can be biased. The detrended fluctuation analysis (DFA) as a fractal dimension method was applied to EEG to assess the DOA [18]. Recently, [19] compared twelve entropy indices as indicators of the DOA that is induced by GABAergic agents and showed that the PE and AE outperformed the others. Other studies have focused on bispectrum-based methods using a higher order spectrum [20–23]. The bispectral approach measures the coupling between the phases of the spectral components. The bispectral index (BIS) has been verified in terms of efficacy and is being used in clinical practice [24]. However, the exact algorithm for the BIS has not been reported and is partially unknown [25]. Furthermore, bispectrum analysis, which is the core descriptor of the BIS, requires extensive calculations [26].

This paper demonstrates that the DOC can be quantified using a novel index that utilizes the inequality in the powers of the EEG spectral components. The Gini index, which was originally used for measuring income inequality in economics, is incorporated in the proposed methods, to measure the inequality in EEG waves. To our knowledge, this is the first study showing that the Gini index could be effective in monitoring the DOC. As an indicator of the DOC, the efficacy was determined by examining Spearman's correlation between the proposed measures and the effect-site concentration of propofol with simple calculations.

## 2. Materials and Methods

### 2.1. Subjects

After obtaining the approval of the Asan Medical Center's Review Board and written informed consent, thirteen volunteers were enrolled in the study. The subjects were aged over 20 years and were previously healthy with no abnormal laboratory results.

### 2.2. EEG Recordings

The EEG was recorded using a QEEG-8 system (LAXTHA Inc., Daejeon, Korea) with seven channels of frontoparietal montages (Fp1, Fp2, F3, F4, P3, P4, and Cz referred to A2 of the international 10–20 system) and digitized at a frequency of 256 Hz and 16 bits of precision. The EEG was continuously recorded from 5 min before the start to 60 min after the end of the anesthetics infusion. A ninth-order Butterworth filter was used to remove the frequencies above 48 Hz from the EEG signals. In our study, analyses use data from channel F3.

### 2.3. Blood Sample Acquisition

Microemulsion propofol (Aquafol-MCT™, Daewon Pharm. Co. Ltd., Seoul, Korea) was used as the general anesthetic [27]. When the volunteers arrived at the operating theatre, electrocardiography, pulse oximetry, end-tidal carbon dioxide partial pressure, and noninvasive blood pressure monitoring was started and EEG electrodes were applied. An 18 G angiocath was placed at the vein for propofol infusion, and a 20 G angiocath was placed in the contralateral radial artery for frequent sampling. The volunteers were preoxygenated with 100% oxygen and then a facial mask with 4 L/min of oxygen was applied. Continuous infusion of intravenous propofol was maintained for 60 min at a fixed rate of 12 mg/kg/h. Blood samples of 4 mL were acquired from the artery and vein at preset intervals: immediately before (0 min) and at 0.5, 1, 1.5, 2, 3, 4, 6, 8, 10, 15, 20, 30, 40, 50, 58, 60, 62, 66, 70, 80, 90, 120, 150, 180, 240, 300, 600, 720, and 1200 min after the beginning of propofol infusion. Additionally, samples were collected at the loss of consciousness (LOC) and recovery of consciousness (ROC). The LOC was assessed by verbally instructing the subjects to close their eyes immediately after the start of propofol infusion and at 10 s intervals until the volunteers did not respond. The ROC was evaluated by instructing the volunteers to open their eyes immediately after the end of propofol infusion at 10 s intervals until the subjects responded. Samples were collected in ethylenediaminetetraacetic acid (EDTA) tubes, centrifuged for 10 min at 3500 RPM, and then stored at −70°C until assay. Details of the anesthetic procedure have been previously described in [28].

### 2.4. Conventional Methods

We compared five conventional methods: spectral entropy, permutation entropy, approximate entropy, detrended fluctuation analysis, and SynchFastSlow, which were investigated in recent studies [19, 30]. Conventional frequency domain-based methods have the following process in common. For one epoch of EEG, the spectral component

(1) Xfk=∑nxn·exp⁡−j2πknN,fk=kNfs,k=0,1,…,N−1,

is calculated using the *N*-point discrete Fourier transform (DFT) for the EEG signal amplitude *x*(*n*) at the time point *n* = {0,1,…, *N* − 1}. In (1), *f*_*k*_ and *f*_*s*_ are the corresponding frequency and sampling frequency of the spectral component, respectively, and *k* is the frequency index. If the frequency range is reduced to *k* = {*L*, *L* + 1,…, *H*}, the frequency of the spectral component is bounded by the band of interest, *f*_*L*_–*f*_*H*_ Hz. For example, when the sampling frequency is 256 Hz and the length of the epoch is 5 s, *L* = 66 and *H* = 201 are appropriate for the analysis within the frequency range 13–40 Hz. The power spectrum of the signal *x*(*n*) is calculated from the spectral component as follows:

(2) $$\begin{array}{c} \overset{ˇ}{X}\left(\;f_{k}\;\right)=X\left(\;f_{k}\;\right)\cdot X^{*}\left(\;f_{k}\;\right), \end{array}$$

where *∗* indicates the complex conjugate. The normalized power spectrum is calculated so that the sum of all frequency powers is equal to 1; that is,

(3) $$\begin{array}{c} P\left(\;f_{k}\;\right)=\frac{\overset{ˇ}{X}\left(f_{k}\right)}{\sum_{i}\overset{ˇ}{X}\left(f_{i}\right)}. \end{array}$$

#### 2.4.1. Spectral Entropy (SpE)

The Shannon entropy represents the minimum information quantity that can soundly express all states of a discrete random variable [31]. SpE is defined as the normalized Shannon entropy of the probability of spectral component occurrence when the signal is considered as a stochastic process:

(4) SpEfL–fH Hz=1log⁡H−L+1∑kPfklog⁡1Pfk,k=L,L+1,…,H.

If the signal consists of only one spectral component, the SpE is equal to 0. In contrast, if all spectral components are uniformly distributed, the SpE becomes 1. Generally, SpE_0.8–32 Hz_ and SpE_0.8–47 Hz_ are used for the estimation of the DOC [12].

#### 2.4.2. Permutation Entropy (PE)

Permutation entropy [32] has been proposed as a complexity measure of epileptic EEG [33] and anesthetic EEG [15]. For the EEG signal amplitude *x*(*n*) at the time point *n* = {0,1,…, *N* − 1}, the vectors **u**_*i*_ are defined as

(5) ui=xixi+τ⋯xi+mτ,i=0,1,…,N−mτ,

where *τ* is the time delay between samples and *m* is the embedding dimension. Then, **u**_*i*_ is expressed in the nondecreasing order:

(6) $$\begin{array}{c} {\hat{u}}_{i}=\left[\begin{array}{c} x\left(i+j_{1}\tau \right)\leq x\left(i+j_{2}\tau \right)\leq \cdots \leq x\left(i+j_{m}\tau \right) \end{array}\right]. \end{array}$$

Each vector ${\hat{u}}_{i}$ is mapped onto one of the *m*! permutation patterns. Then, the probability of the *j*th pattern occurring, *p*_*j*_, is

(7) $$\begin{array}{c} p_{j}=\frac{n_{j}}{\sum_{j}n_{j}},\;j=\left\{1,2,\ldots ,m!\right\}, \end{array}$$

where *n*_*j*_ is the number of occurrences of the *j*th permutation. In [32], PE is defined as −∑_*j*_(*p*_*j*_log⁡*p*_*j*_)/log⁡(*m*!) and, in [15], it is modified as

(8) $$\begin{array}{c} PE=-\frac{\sum_{j}p_{j}\log{p_{j}}_{\tau =1}+\sum_{j}p_{j}\log{p_{j}}_{\tau =2}}{\log{\left(m+1\right)}^{2}} \end{array}$$

to include both slow and fast EEG oscillations. We used *m* = 3, as recommended in [15]. Because of the extensive repetition of ordinal patterns in slow waves, PE is dominated by the proportion of higher EEG frequencies.

#### 2.4.3. Approximate Entropy (AE)

Approximate entropy, as an approximation of the Kolmogorov-Sinai entropy, quantifies the randomness of a time series signal and has been evaluated [16] for application in the analysis of EEG signals associated with anesthetic effects. The vectors **u**_*i*_ are defined as

(9) ui=xixi+1⋯xi+m−1,i=0,1,…,N−m,

where *m* is the embedding dimension that determines the dimension of the phase space. The fraction that expresses whether **u**_*j*_ is within the filtering distance *r* of **u**_*i*_ is defined as

(10) $$\begin{array}{c} C_{i}^{m}\left(\;r\;\right)=\frac{number\;\;of\;\;such\;\;j\;\;that\;\;\max\left|u_{i}-u_{j}\right|\leq r}{N-m+1}. \end{array}$$

The AE is defined by

(11) $$\begin{array}{c} AE\left(\;m,r\;\right)=\varphi^{m}\left(\;r\;\right)-\varphi^{m+1}\left(\;r\;\right), \end{array}$$

where

(12) $$\begin{array}{c} \varphi^{m}\left(\;r\;\right)=\frac{1}{N-m+1}\overset{N-m+1}{\underset{i=1}{\sum }}\ln C_{i}^{m}\left(\;r\;\right). \end{array}$$

The AE is known to decrease with increasing anesthetic concentration. We used the parameter set *N* = 1024, *m* = 2, *r* = 0.2 × {SD  of  **u**_*i*_} as recommended in [16].

#### 2.4.4. Detrended Fluctuation Analysis Exponent (DFA)

Reference [18] used the DFA technique to study the scaling behavior of the EEG. For EEG signal of length *N*, the integrated series is defined as

(13) $$\begin{array}{c} D\left(\;k\;\right)=\overset{k}{\underset{i=1}{\sum }}\left(\;x\left(\;i\;\right)-x_{average}\;\right),\;k=1,\ldots ,N. \end{array}$$

Then, *D*(*k*) is divided into nonoverlapping segments of length *n*, and *D*_*n*_(*k*) is the linear regression of the segment. The root mean square fluctuation of *D*(*k*) from the trend is

(14) $$\begin{array}{c} F_{n}=\sqrt{\frac{1}{N}\overset{N}{\underset{i=1}{\sum }}{\left(D\left(k\right)-D_{n}\left(k\right)\right)}^{2}}. \end{array}$$

Exponent *α* is the slope of the line in log-log representation, by using the linear regression of *F*_*n*_ in function of *n*, *F* = *n* ^*α*^. We calculated *α*_3_ with the segment length, *n*, associated with 6.7–157.8 ms as recommended in [18].

#### 2.4.5. SynchFastSlow

The bispectrum approach is a method of measuring the degree of phase coupling between two spectral components contained in a signal. Unlike the SpE, which only uses the power spectrum, the phase information is not ignored. The bispectrum magnitude is defined as

(15) BfL–fH Hzfi,fj=∑lXlfiXlfjXl∗fi+fj,fi,fj∈fL,fH,

where *X*_*l*_ is the spectral component of the *l*th epoch. Although the relationship between the LOC and phase coupling has not been clarified, an increase in phase coupling has been observed during anesthesia. The bispectrum has been used to estimate the degree of anesthesia in clinical trials [25]. The bispectral index (BIS), a common indicator of the DOC, uses SFS which incorporates the bispectrum [20, 34]:

(16) $$\begin{array}{c} SFS=\log\frac{\sum_{f_{i},f_{j}}B_{0.5\text{–}47\;Hz}\left(f_{i},f_{j}\right)}{\sum_{f_{i},f_{j}}B_{40\text{–}47\;Hz}\left(f_{i},f_{j}\right)}. \end{array}$$

This is approximately the logarithmic ratio of the bispectrum magnitude values in the delta, theta, alpha, beta, and gamma band versus that in the gamma band only.

### 2.5. Proposed Methods

#### 2.5.1. Spectral Gini Index (SpG)

The Gini index [35] was originally used to quantify income inequality in the field of economics. If the income level of the *i*th (*i* = {1,2,…, *N*}) house is *x*_*i*_, the Gini index is calculated using the following equation [36]:

(17) $$\begin{array}{c} G\left(\;x\;\right)=\frac{\sum_{i=1}^{N}\sum_{j=1}^{N}\left|x_{i}-x_{j}\right|}{2N\sum_{i=1}^{N}x_{i}}. \end{array}$$

If the incomes of all houses are equal, that is, *x*_1_ = *x*_2_ = ⋯ = *x*_*N*_, the Gini index becomes 0. Additionally, when only one house has income, that is, *x*_1_ > *x*_2_ = ⋯ = *x*_*N*_ = 0, the income inequality is maximum and the Gini index is equal to 1.

The proposed method incorporates the Gini index to quantify the inequality between power spectra in the range of interest, *f*_*L*_–*f*_*H*_ Hz. If each frequency of the power spectrum of the EEG signal is considered as an individual house and the power of the corresponding frequency is considered as the house income, we can quantify the spectral inequality in terms of the Gini index. Therefore, the proposed spectral Gini index (SpG) is expressed as

(18) $$\begin{array}{c} SpG_{f_{L}\text{–}f_{H}\;Hz}=\frac{\sum_{i=1}^{N}\sum_{j=1}^{N}\left|\overset{ˇ}{X}\left(f_{i}\right)-\overset{ˇ}{X}\left(f_{j}\right)\right|}{2\left(H-L+1\right)\sum_{i=L}^{H}\overset{ˇ}{X}\left(f_{i}\right)}. \end{array}$$

The SpG can measure the inequality in the spectral powers of the signal. For example, in a white Gaussian random signal, all spectral components have equal powers; thus the SpG becomes 0. In contrast, for a signal focused on a certain spectral component, the SpG approaches 1.

#### 2.5.2. Binarized Spectral Gini Index (BSpG)

Here, we introduce the Gini index of the binarized spectrum. Firstly, we define the binarized power spectrum $\tilde{X}$:

(19) $$\begin{array}{c} \tilde{X}\left(\;f_{k}\;\right)=\left\{\begin{array}{cc} 1, & \overset{ˇ}{X}\left(f_{k}\right)>\alpha \\ 0, & \overset{ˇ}{X}\left(f_{k}\right)\leq \alpha , \end{array}\right. \end{array}$$

where *α* is a parameter proportional to the average power of the EEG before injection. Then, the BSpG is defined as

(20) $$\begin{array}{c} BSpG_{f_{L}\text{–}f_{H}\;Hz}=\frac{\sum_{i=1}^{N}\sum_{j=1}^{N}\left|\tilde{X}\left(f_{i}\right)-\tilde{X}\left(f_{j}\right)\right|}{2\left(H-L+1\right)\sum_{i=L}^{H}\tilde{X}\left(f_{i}\right)}. \end{array}$$

The advantage of the BSpG is that its calculation is very simple. Considering a total of *N*  (*N* = *H* − *L* + 1) values, if *M*  (*M* ≤ *N*) values are equal to 0,  (20) is simplified as follows:

(21) $$\begin{array}{c} BSpG_{f_{L}\text{–}f_{H}\;Hz}=\frac{2M\left(N-M\right)}{2N\left(N-M\right)}=\frac{M}{N}. \end{array}$$

**Figure 1:**
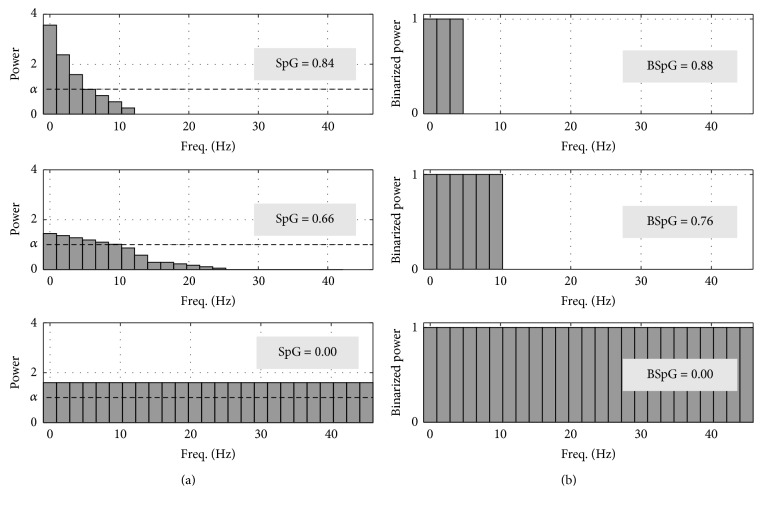
SpG and BSpG for various power spectra: (a) SpG and (b) BSpG. Both SpG and BSpG can measure the inequality of spectral distributions.

Figures 1(a) and 1(b) show an example of the SpG and BSpG for various power spectra with different distributions. The power spectrum at the top of Figure 1(a) is mostly concentrated below 10 Hz, showing an inequality with an SpG value of 0.84. In comparison, the spectrum in the middle exhibits less inequality and has a decreased SpG value of 0.66. The power spectrum at the bottom is uniform; therefore, the SpG is 0.00, the minimum value. The power spectra of Figure 1(b) show the binarized power spectra and the relevant BSpG. As shown in the upper left spectrum, the percentage of spectral components with powers below the threshold is 88%, and the BSpG value is 0.88. For the middle spectrum which exhibits less inequality, the BSpG has a smaller value. Finally, for the spectrum that shows perfect equality, the BSpG is equal to 0.00, as in the case of SpG. Thus, we consider that both the SpG and the BSpG can measure the inequality of spectral distributions.

### 2.6. Population Pharmacokinetic Analysis

A population pharmacokinetic analysis was performed with NONMEM VII level 3 (ICON Development Solutions, Ellicott City, MD, USA). Interindividual random variabilities of pharmacokinetic parameters were estimated assuming a log-normal distribution. Diagonal matrices were estimated for the various distributions of *η*, where *η* represented interindividual random variability with a mean of zero and a variance of *ω* ^2^. Additive, constant coefficient of variation, and combined additive and constant coefficient of variation residual error models were evaluated during the model building process. NONMEM computed the minimum objective function value (OFV), a statistic equivalent to the −2log⁡ likelihood of the model. An *α* level of 0.05, which corresponds to a reduction in the OFV of 3.84 (Chi-square distribution, degree of freedom = 1, *p* < 0.05), was used to distinguish between hierarchical models [37]. One-, two-, and three-compartment disposition models with first-order elimination were tested. The covariates analysed were age, sex (0 = male, 1 = female), weight, height, body surface area [38], body mass index, ideal body weight [39], and lean body mass [29]. Nonparametric bootstrap analysis served to validate the models internally (fit4NM 3.5.1, Eun-Kyung Lee and Gyu-Jeong Noh, http://cran.r-project.org/web/packages/fit4NM/index.html, last access: Oct 11, 2011) [40].

### 2.7. EEG Data Selection

The selection criteria for each EEG index used in this study were as follows: (1) every 30 s during the first 10 min, every 1 min during the second 60 min, and after the beginning of the propofol infusion and (2) every 30 s during the first 30 min, every 1 min during the second 20 min, and every 2 min during the third 20 min, after the termination of the propofol infusion [41].

### 2.8. Population Pharmacodynamic Analysis

A sequential modeling approach with post hoc pharmacokinetic estimates was used to derive the population pharmacodynamic parameters. Dissociation between the concentration of propofol and effect of propofol on central nervous system (EEG indices) was linked with an effect compartment. The relationship between the effect-site concentration (*C*_e_) of propofol and EEG indices was evaluated using a sigmoid *E*_max_ model:

(22) $$\begin{array}{c} E=E_{0}+\left(\;E_{max}-E_{0}\;\right)\frac{C_{e}^{\gamma }}{C_{e_{50}}^{\gamma }+C_{e}^{\gamma }}, \end{array}$$

where *E* is each EEG index value, *E*_0_ is the baseline EEG index value when no drug was present, *E*_max_ is the maximum possible drug effect on the EEG index, *C*_e_ is the calculated effect-site concentration of propofol, *C*_e_50__ is the effect-site concentration associated with 50% of the maximal drug effect on EEG index, and *γ* is the steepness of the effect-site concentration versus EEG index relationship.

### 2.9. Statistics

Prediction probability (*P*_*K*_) was assessed as described by Smith and colleagues [42]. We calculated *P*_*K*_ values using Somers' *D* cross-tabulation statistic on SPSS, which was then transformed from the −1 to 1 scale of Somers' *D* to 0 to 1 scale of *P*_*K*_ as *P*_*K*_ = 1 − (1−|Somers'  *D*|) × 2^−1^. The EEG indices and *C*_e_ were set as the dependent and independent variables, respectively. Prediction probabilities were calculated using the full measurement set. The SE of each *P*_*K*_ was calculated as (SE of Somers' *D*) × 2^−1^.

## 3. Results and Discussion

To demonstrate the efficacy of the conventional indices (SpE, PE, AE, DFA, and SFS) and the proposed ones (SpG, BSpG), all indices were applied to the EEG signal obtained during a period of approximately 130 min that included the preanesthesia stage, the anesthesia stage, including the LOC, and the recovery stage including the ROC. The EEG signal was equally divided into 10 s epochs. To prevent spectrum distortion due to the DFT procedure, the Blackman window was applied to each epoch, and the fast Fourier transform (FFT) was used to obtain the spectral components. To maintain time continuity, each epoch was overlapped for 5 s. Therefore, all index values were calculated every 5 s. All the index parameters were optimized with each index's best condition. The smoothing rate for all the indices was 30 s. The values obtained through this process were compared with the effect-site concentration of propofol to determine how well they reflected the DOC.

**Figure 2:**
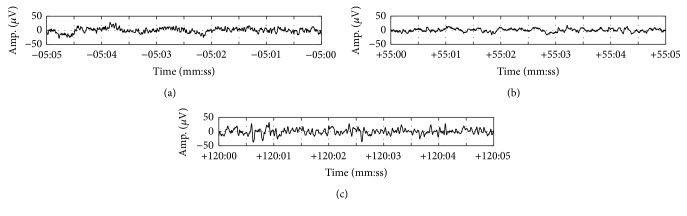
Examples of 5 s long EEG signals for (a) the “awake” state before the beginning of the anesthetic infusion, (b) the “anesthetized” state with the maximum plasma concentration of propofol, and (c) the state after the ROC. As the depth of consciousness increases, EEG signal exhibits slowly changing activities.

Figures 2(a)–2(c) show 5 s EEG signals extracted from three different anesthesia states. Anesthesia causes characteristic changes in the spectral component of EEG signal. As the depth of consciousness increases, EEG signal exhibits decreased high spectral component.  Figure 2(a) displays “awake” state EEG signal for the period before the anesthetic infusion.  Figure 2(b) shows the EEG signal in deep general anesthesia with the maximum plasma concentration of propofol.  Figure 2(c) illustrates the EEG signal for the period after the ROC.

**Figure 3:**
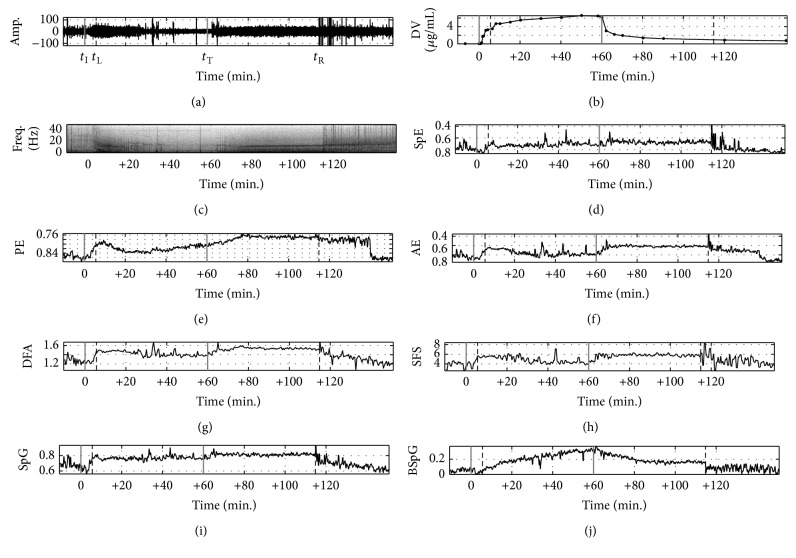
Example of the relationship between the changes in the EEG signal and various methods during general anesthesia with propofol. (a) EEG signal, (b) plasma concentration of propofol, and (c) spectrogram. (d), (e), (f), (g), (h), (i), and (j) show the SpE: spectral entropy, PE: permutation entropy, AE: approximate entropy, DFA: detrended fluctuation analysis exponent, SFS: SynchFastSlow, SpG: spectral Gini index, and BSpG: binarized spectral Gini index, respectively.

Figure 3 shows EEG signals obtained during the entire anesthesia procedure, the plasma concentration of propofol, the spectrogram of the EEG, and the conventional and the proposed indices.  Figure 3(a) depicts the unprocessed EEG signal recorded by channel F3. The plasma concentration of propofol (*μ*g/mL) in Figure 3(b) is included for the standard evaluation of the anesthesia depth estimation. On the horizontal axis, *t*_I_ represents the initial time of injection and is set to the timeline standard of 0 min 0 s. On the other hand, *t*_T_ is the time of the injection termination and extends to 60 min after *t*_I_. The two vertical dashed lines, *t*_L_ and *t*_R_, represent the time of the LOC and the ROC, respectively.  Figure 3(c) displays the results of the time-frequency analysis and shows the change of the power distribution in the 0–47 Hz as a function of time. The power is weakly concentrated only in the 8–12 Hz band before *t*_I_ but becomes extending across the 0.5–3 Hz and the 13–47 Hz bands after *t*_I_. In other words, the power distribution is relatively uniform in a normal conscious state but the spectral inequality increases and the power concentrates on certain bands after anesthesia induction. Between *t*_L_ and *t*_I_ + 60 min, when the plasma concentration of propofol reaches its peak, the power is concentrated on the 0.5–3 Hz band, while that in the 30–45 Hz band slowly decreases. That is, the inequality in power increases further. Immediately after *t*_T_, no noticeable change is observed in the 0.5–3 Hz band but an increase in power is shown in the 8–13 Hz band. After *t*_R_, the power concentrated on the 0.5–3 Hz band mostly disappears, and the power spectrum pattern became similar to that before *t*_I_. Figures 3(d)–3(h) show the results of the conventional and the proposed indices. For the indices in Figures 3(d)–3(f), lower values indicate deeper anesthesia. Therefore, in contrast to the other indices, the vertical axis is reversed for comparison convenience. SpE_0.8–47 Hz_ is shown in Figure 3(d). The SpE index during anesthesia does not show significant change compared with that before anesthesia. The PE and AE presented in Figures 3(e) and 3(f) do not change proportionally to the plasma concentration of propofol; they increase immediately after *t*_L_ and decrease directly after *t*_T_. The DFA and the SFS in Figures 3(g) and 3(h) and the AE exhibit similar characteristics.  Figure 3(i) displays the variation of the proposed index, SpG. During anesthesia, the SpG looks similar to the SpE but smaller fluctuations after *t*_R_.  Figure 3(j) shows the other proposed index, BSpG, in the 0.8–47 Hz band. The threshold for the binarized power spectrum was set to 2% of the average power in the 0.8–47 Hz band before *t*_I_. The values from *t*_I_ to *t*_T_ gradually increase, appropriately reflecting the DOC during anesthesia. In addition, after *t*_T_, the BSpG gradually decreases to a normal state level. This means that BSpG_0.8–47 Hz_ can appropriately reflect the DOC during recovery. Additionally, sharp changes can be observed near *t*_L_ and *t*_R_ that are clearly related to the LOC and ROC points.

The anesthesia stages were divided into four different periods to perform statistical analysis of the test results: the “induction” stage was set to the period from *t*_I_ to *t*_L_; the “deep hypnosis” stage was the 20 min interval before *t*_T_; the “awakened” stage was the 20 min interval before *t*_R_; the “post” stage was the 20 min interval after *t*_T_ + 40 min. The duration of the “induction” stage ranged from 2 min to 13 min, and all the other stages were considered to last for 20 min.

**Figure 4:**
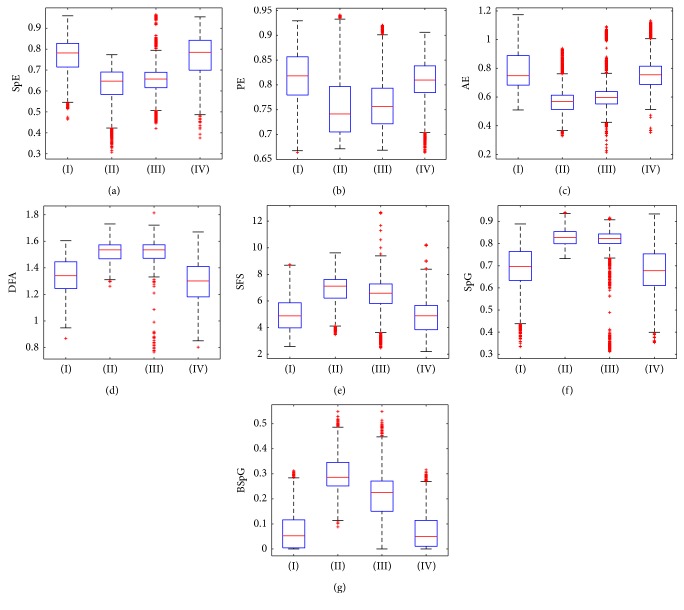
Boxplots of the conventional and proposed indices at induction (I), deep hypnosis (II), awakened (III), and post (IV) stages. (a) SpE, (b) PE, (c) AE, (d) DFA, (e) SFS, (f) SpG, and (g) BSpG.

Table 1 shows the statistic values of the conventional indices (SpE, PE, AE, DFA, and SFS) and the proposed indices (SpG and BSpG) during the four anesthetic stages (induction, deep hypnosis, awakened, and post) for all subjects. Data are expressed as median and 2.5–97.5 percentile. The boxplots of all indices at induction (I), deep hypnosis (II), awakened (III), and post (IV) stages are shown in Figure 4. Both the SpG and BSpG could distinguish between “deep hypnosis” and “induction/post” stages.

### 3.1. Population Pharmacokinetic Analysis

In total, 449 plasma concentration measurements from 15 healthy volunteers were used to characterize the pharmacokinetics of propofol. A three-compartment mammillary model best described the pharmacokinetics of propofol. Lean body mass was a significant covariate for the central volume of distribution (*V*_*d*_) (see (23)), and it resulted in improvement in the OFV (7.22, *p* = 0.007, degree of freedom = 1), compared with the basic model (number of model parameters = 10).

(23) $$\begin{array}{c} V_{1}=10.3+{\left(\;\frac{LBM}{45}\;\right)}^{6.63}. \end{array}$$

Body weight was a significant covariate for the metabolic clearance of propofol (see (24)) and resulted in an improvement in OFV (9.18, *p* = 0.002, df = 1) compared with the OFV of a pharmacokinetic mode that included LBM as a covariate for the central *V*_*d*_ (number of model parameters = 11).

(24) $$\begin{array}{c} Cl=0.217+{\left(\;\frac{\text{WT}}{63}\;\right)}^{1.02}. \end{array}$$

Table 3 presents the population pharmacokinetic parameter estimates and the results of nonparametric bootstrap replicates of the final pharmacokinetic model of propofol.

### 3.2. Population Pharmacodynamic Analysis

A total of 2147 EEG data points were used to determine the pharmacodynamic characteristics of each EEG index. A sigmoid *E*_max_ model well described the time course of observed EEG indices values. Population pharmacodynamic parameter estimates and interindividual variability of the pharmacodynamic models are shown in Table 4.

### 3.3. Prediction Probability and Spearman's Correlation Coefficient

The *P*_*k*_ values and Spearman's correlation coefficients of the EEG indices are shown in Table 2. Those values were largest in the BSpG (*R* = 0.770, *p* < 0.001), which indicates that the BSpG is appropriate for the assessment of the propofol effect on the electroencephalogram.

### 3.4. Computational Complexity of SpG and BSpG

To evaluate the computational complexity of the proposed methods, Table 5 provides the running time of the conventional and proposed methods for the examined dataset. The test was performed using a computer with Intel Core i5-4460 @3.20 GHz and 8 GB RAM. The data were processed offline using the Matlab R2015a (MathWorks Inc., MA, USA) software. The results show that the computational costs of the SpG and BSpG were significantly lower than those of the other methods.

### 3.5. Comparison Remark with the Conventional Approaches

The Gini index has scale independence and population independence and is the most popular metric for operationalizing income inequality [43] and is highly sensitive to inequalities in the middle values of the input data [44]. Moreover, the Gini index is not based on any model of a probability distribution unlike the approaches based on entropy. Therefore, not an assumption of statistical characteristics of EEG nor sample parameter is needed.

The SpG and the entropy measures, especially the SpE, are alike in that the inequality in power spectrum is characterized. However, the sensitivity to the change of spectral variation differs. For a normalized power spectrum $x=\left[\begin{array}{cccc} x_{1} & x_{2} & \cdots & x_{N} \end{array}\right]$ where ∑_*i*_ *x*_*i*_ = 1 and *x*_*i*_ < 1, the SpE is −∑_*i*=1_^*N*^ *x*_*i*_log⁡*x*_*i*_. If one spectral power component *x*_*a*_ is multiplied by *A*, then the normalized power spectrum is changed as

(25) x′=x11+A−1xa⋯Axa1+A−1xa⋯xN1+A−1xa,

the SpE becomes

(26) SpE=−∑i=1Nxi1+A−1xalog⁡xi1+A−1xa=−11+A−1xa·∑i=1Nxilog⁡xi−xilog⁡1+A−1xa=−11+A−1xa∑i=1Nxilog⁡xi−log⁡1+A−1xa=log⁡1+A−1xa−∑i=1Nxilog⁡xi1+A−1xa,

and the SpG is expressed as

(27) $$\begin{array}{c} SpG=\frac{\sum_{i=1}^{N}\sum_{j=1}^{N}\left|x_{i}-x_{j}\right|+\left(N-1\right)\left|A-1\right|x_{a}}{2N\left(\sum_{i=1}^{N}x_{i}+\left|A-1\right|x_{a}\right)}. \end{array}$$

**Figure 5:**
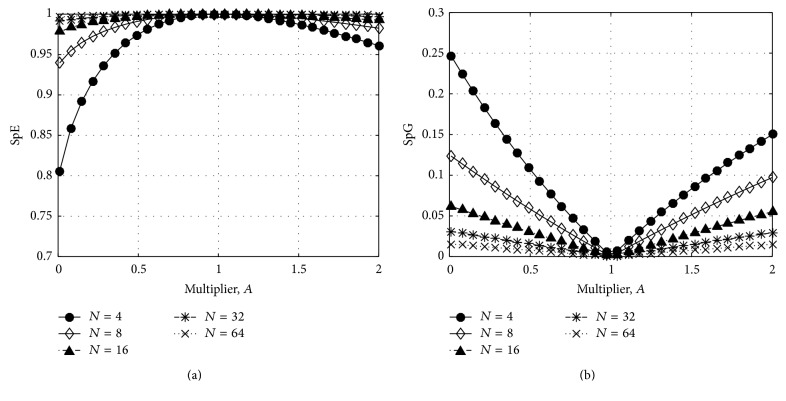
Comparison of the change of index between the SpE and SpG: (a) SpE and (b) SpG. The SpG can reflect the variation more proportionally according to the multiplier, *A*.

To illustrate the change of the SpE and SpG according to *A*, we set $x=\left[\begin{array}{cccc} 1/N & 1/N & \cdots & 1/N \end{array}\right]$ and *N* = 4,8, 16,32,64.  Figure 5 compares the SpE with SpE. The SpG can reflect the spectral variation more proportionally.

## 4. Conclusions

In this study, the Gini index which was originally used for measuring income inequality in economics was applied to EEG spectral analysis to estimate the DOC. The proposed index requires no sample parameter estimation and thus no probability distribution function, unlike the conventional indices. In addition, because its computational complexity is low, the index has the advantage of real-time implementation even with multiple EEG channels. We have demonstrated that the proposed indices exhibit a higher correlation with the effect-site concentration of propofol compared with the conventional ones.

In deep anesthesia, a certain EEG pattern can be observed owing to cerebral mechanisms. Thus, a method that detects this pattern is considered to be very important [30, 45–47]. Although the proposed index alone cannot fully quantify the DOC, it can play a valuable role in the quantification of the DOC. Further studies may be needed to examine how well the proposed method reflects the DOC in a slow injection. Moreover, the role of the SpG as a measure of the DOC for other anesthetics, such as ketamine or sevoflurane, requires further investigation.

## Figures and Tables

**Table 1:** The statistics of indices during four anesthetic stages (median (2.5–97.5%)).

Index	Induction	Deep hypnosis	Awakened	Post
SpE	0.78 (0.71–0.93)	0.65 (0.58–0.74)	0.66 (0.62–0.94)	0.79 (0.70–0.92)
PE	0.82 (0.78–0.91)	0.74 (0.71–0.94)	0.76 (0.72–0.88)	0.81 (0.78–0.89)
AE	0.75 (0.68–1.07)	0.57 (0.51–0.83)	0.60 (0.55–1.01)	0.75 (0.69–1.07)
DFA	1.34 (1.24–1.59)	1.53 (1.47–1.64)	1.54 (1.47–1.65)	1.30 (1.18–1.57)
SFS	4.88 (3.98–7.39)	7.12 (6.21–8.60)	6.58 (5.81–8.60)	4.88 (3.84–7.19)
SpG	0.70 (0.63–0.85)	0.83 (0.80–0.92)	0.82 (0.80–0.89)	0.68 (0.61–0.87)
BSpG	0.05 (0.00–0.27)	0.29 (0.25–0.45)	0.23 (0.15–0.37)	0.05 (0.01–0.24)

Induction: [*t*_I_, *t*_L_], deep hypnosis: [*t*_T_ − 20 min, *t*_T_], awakened: [*t*_R_ − 20 min, *t*_R_], and post: [*t*_T_ + 40 min, *t*_T_ + 60 min].

**Table 2:** Prediction probability (*P*_*K*_) values and Spearman's correlation coefficients of the EEG indices.

Index	*P*_*K*_ (SE, 95% CI)	Spearman's corr. coeff.
SpE	0.7129 (0.0060, 0.7012–0.7246)	−0.602^*∗*^
PE	0.6349 (0.0078, 0.6195–0.6503)	−0.359^*∗*^
AE	0.7264 (0.0067, 0.7133–0.7396)	−0.606^*∗*^
DFA	0.6930 (0.0067, 0.6799–0.7061)	0.557^*∗*^
SFS	0.6636 (0.0072, 0.6495–0.6777)	0.463^*∗*^
SpG	0.7244 (0.0059, 0.7128–0.7360)	0.633^*∗*^
BSpG	0.7837 (0.0043, 0.7752–0.7922)	0.770^*∗*^

SE: standard error; CI: confidence interval; ^*∗*^ *p* < 0.001.

**Table 3:** Population pharmacokinetic parameter estimates, interindividual variability, and median parameter values (2.5–97.5%) of the nonparametric bootstrap replicates of the final pharmacokinetic model of propofol.

Parameters	Unit		Estimates (RSE, %)	CV (%)	Median (2.5–97.5%)
$V_{1}=\theta_{1}+{\left(\frac{LBM}{45}\right)}^{\theta_{2}}$	L	*θ*_1_	10.3 (6.4)	10.7	10.3 (9.3–11.7)
		*θ*_2_	6.63 (15.7)		6.4 (4.3–10.8)
*V*_2_	L		75.3 (9.4)	—	75.3 (62.6–88.4)
*V*_3_	L		846 (41.0)	—	861 (505.9–1600)
$Cl=\theta_{3}+{\left(\frac{WT}{58}\right)}^{\theta_{4}}$	L/min	*θ*_3_	0.217 (49.3)	11.2	0.214 (0.00002–0.36)
		*θ*_4_	1.02 (43.3)		0.99 (0.0005–1.79)
*Q*_1_	L/min		0.928 (6.1)	—	0.928 (0.829–1.05)
*Q*_2_	L/min		0.679 (13.2)	19.3	0.703 (0.569–0.895)
*σ*	—		0.083 (9.8)	—	0.081 (0.066–0.095)

A log-normal distribution of interindividual random variability was assumed. Residual random variability was modeled using constant CV error model. Nonparametric bootstrap analysis was repeated 1000 times. RSE: relative standard error = SE/mean × 100 (%). LBM: lean body mass calculated using the Janmahasatian formula [29], WT: body weight, *V*_1_: central volume of distribution (*V*_*d*_), *V*_2_: rapid peripheral *V*_*d*_, *V*_3_: slow peripheral *V*_*d*_, Cl: metabolic clearance, *Q*_1_: intercompartmental clearance between central and rapid peripheral compartments, and *Q*_2_: intercompartmental clearance between central and slow peripheral compartments.

**Table 4:** Population pharmacokinetic parameter estimates and interindividual variability of the pharmacokinetic models of propofol.

Index	*E*_0_	*E*_max_	*C*_e_50__	*γ*	*k*_e_0__
SpE	0.796 (2.4, 9.2)	0.607 (2.9, 13.3)	1.47 (0.5, 52.6)	6.54 (3.0, 175.5)	0.089 (0.4, 33.5)
PE	0.698 (17.8, 14.4)	0.293 (43.0, 97.4)	1.18 (22.4, 44.4)	2.99 (46.5, 57.5)	0.1 (28.6, 70.1)
AE	0.852 (4.9, 14.7)	0.278 (1.5, 71.0)	1.12 (0.3, 52.1)	5.52 (8.0, 147.3)	0.117 (2.0, 33.0)
DFA	1.250 (2.4, 9.6)	1.530 (0.9, 3.1)	1.33 (12.0, 33.5)	7.73 (6.2, 150.0)	0.105 (10.3, 53.7)
SFS	4.17 (4.5, 16.6)	6.81 (4.2, 16.0)	1.1 (11.9, 42.7)	6.55 (18.6, 83.7)	0.146 (14.2, 53.9)
SpG	0.68 (11.3, 17.8)	0.826 (1.3, 3.44)	1.22 (0.3, 43.9)	5.73 (43.1, 198.8)	0.098 (14.4, 45.4)
BSpG	0.037 (28.7, 99.7)	0.367 (4.3, 46.9)	2.88 (10.2, 55.2)	3.85 (5.4, 126.5)	0.081 (5.1, 34.2)

Data are expressed estimate (RSE, % CV). A log-normal distribution of interindividual random variability was assumed. Residual random variability was modeled using additive error model. RSE: relative standard error = SE/mean × 100 (%). SE: standard error.

**Table 5:** Running time (for data collected in time windows of 10 s) of the conventional and the proposed methods.

Running time	SpE	PE	AE	DFA	SFS	SpG	BSpG
Average (ms)	0.09936	55.2425	44.2638	12.7942	2.90514	0.08960	0.08498
SD (ms)	0.00713	2.09184	1.30907	0.2034	0.08407	0.00714	0.00704

SD: standard deviation.
